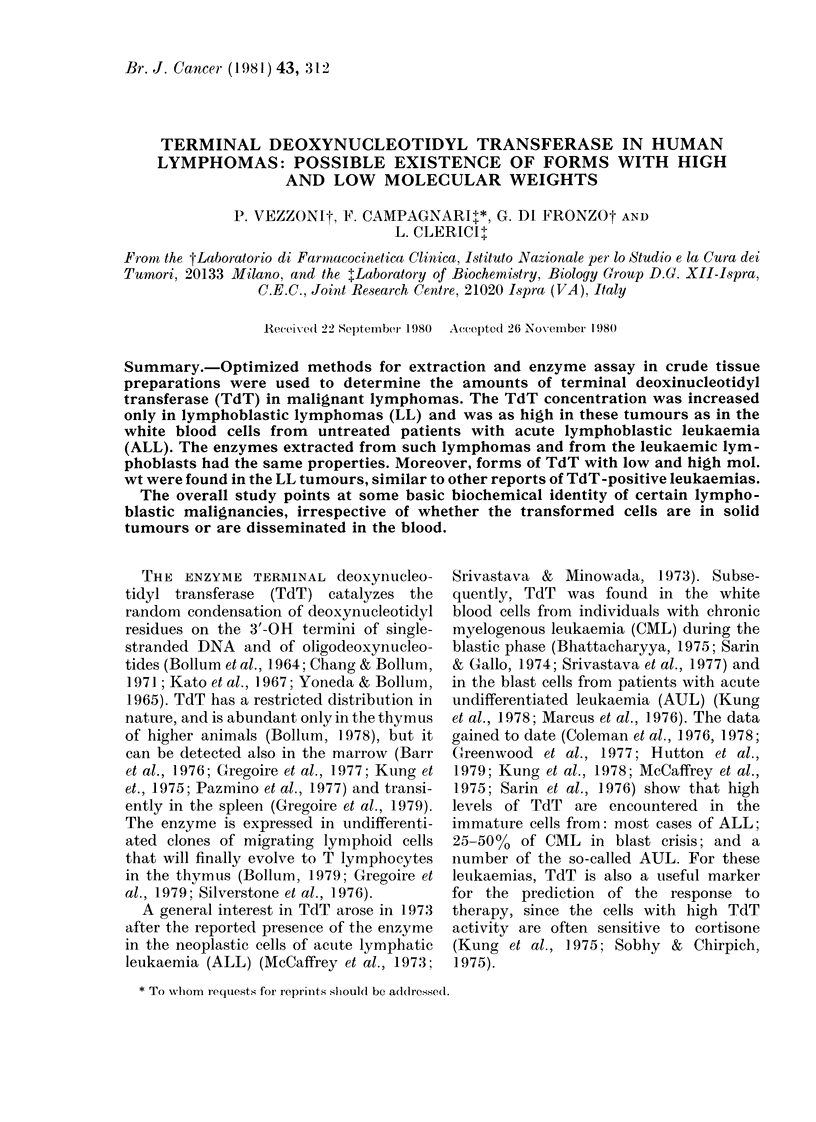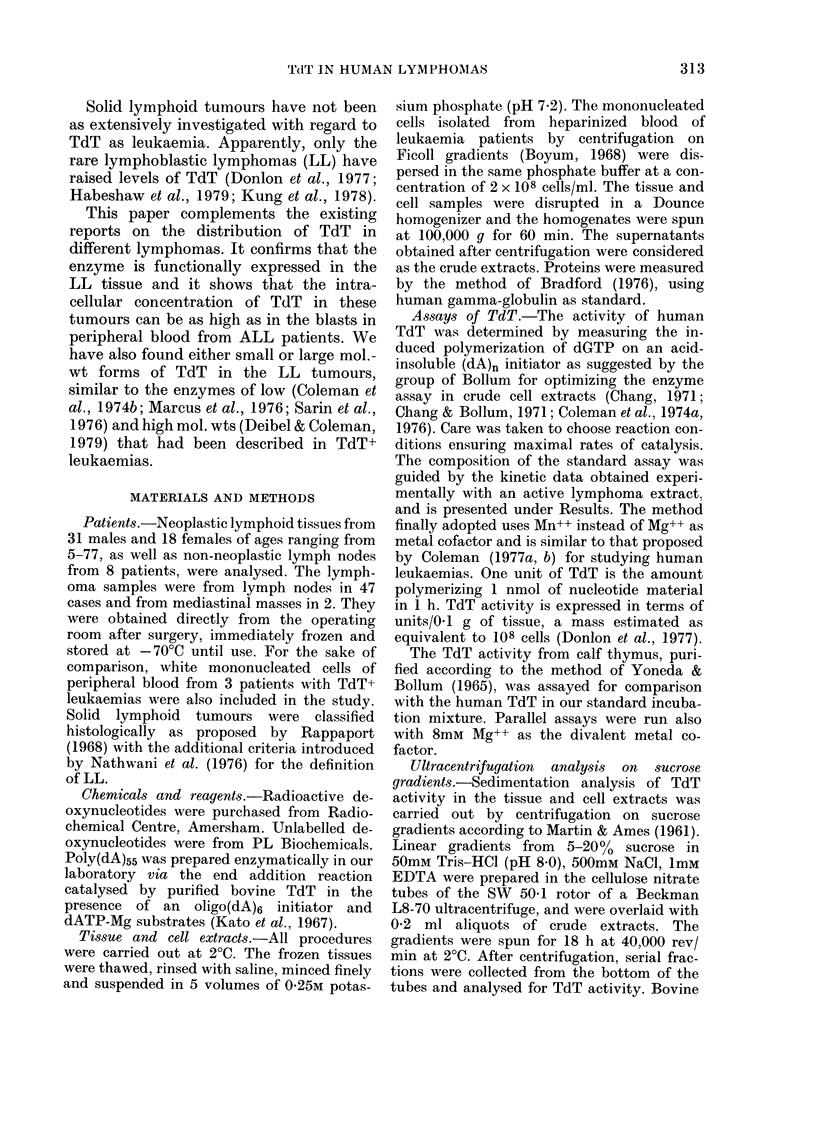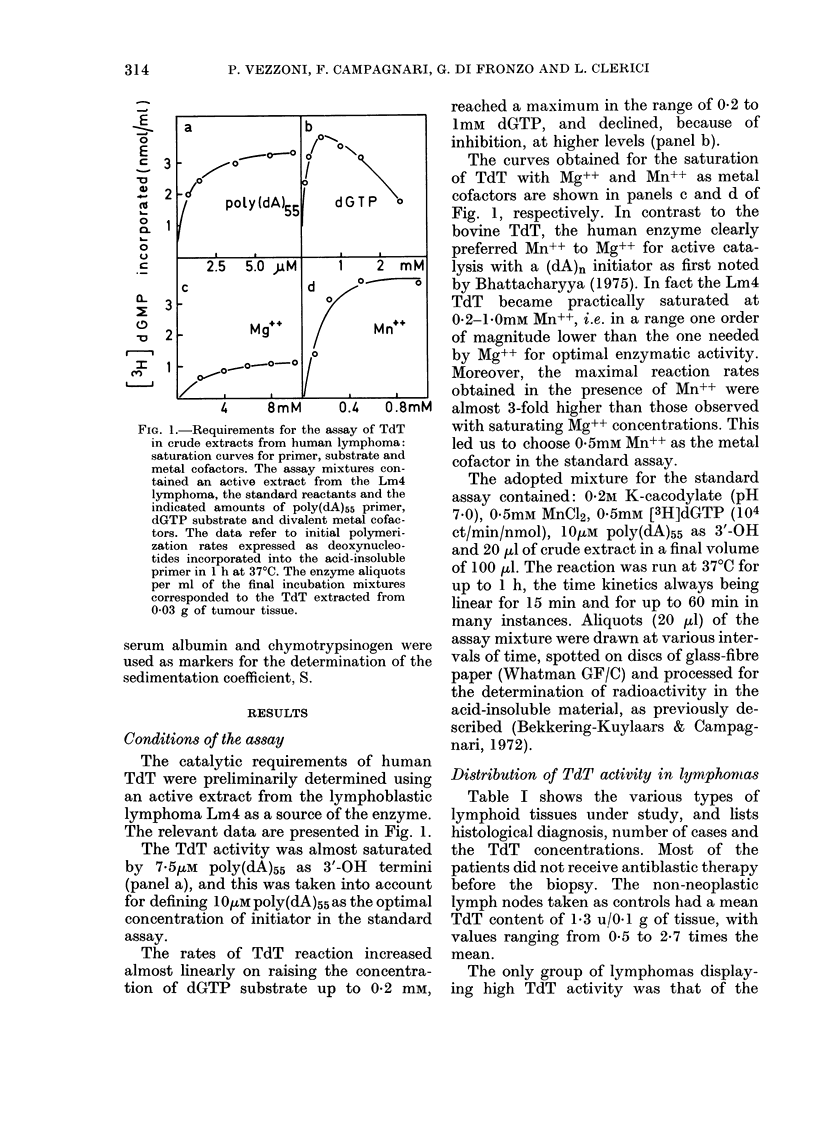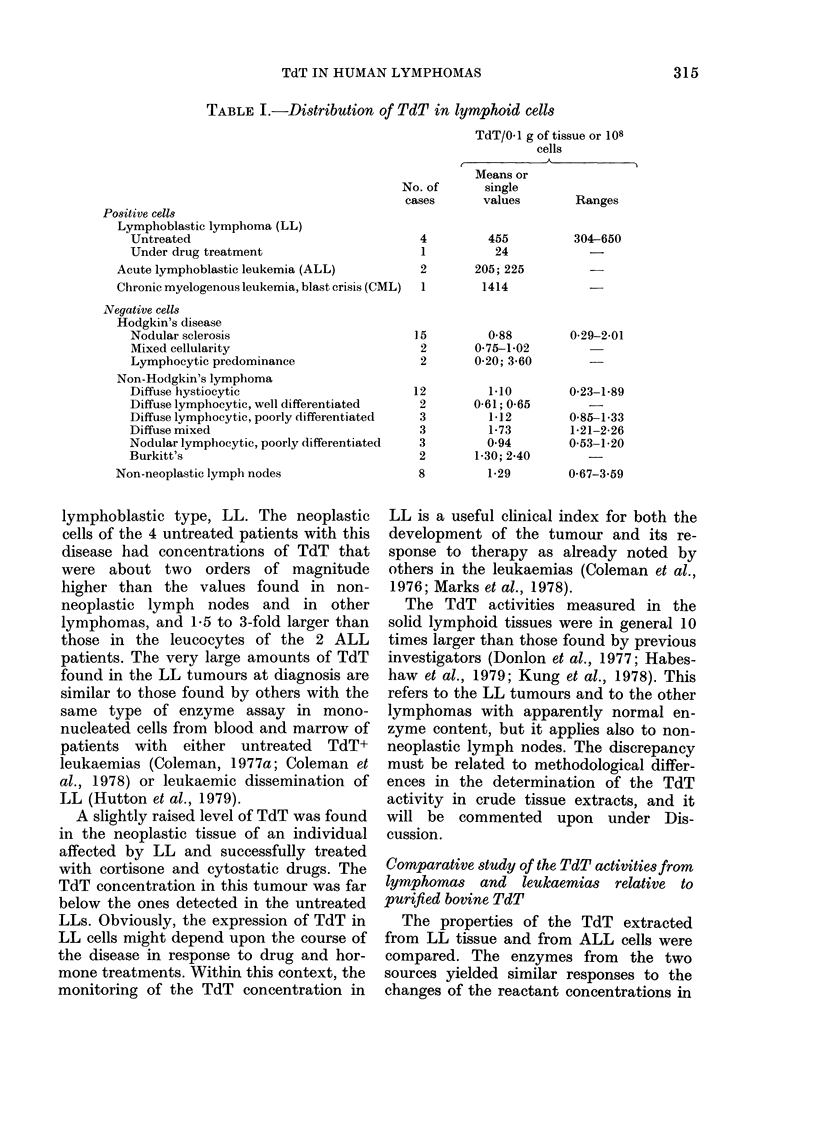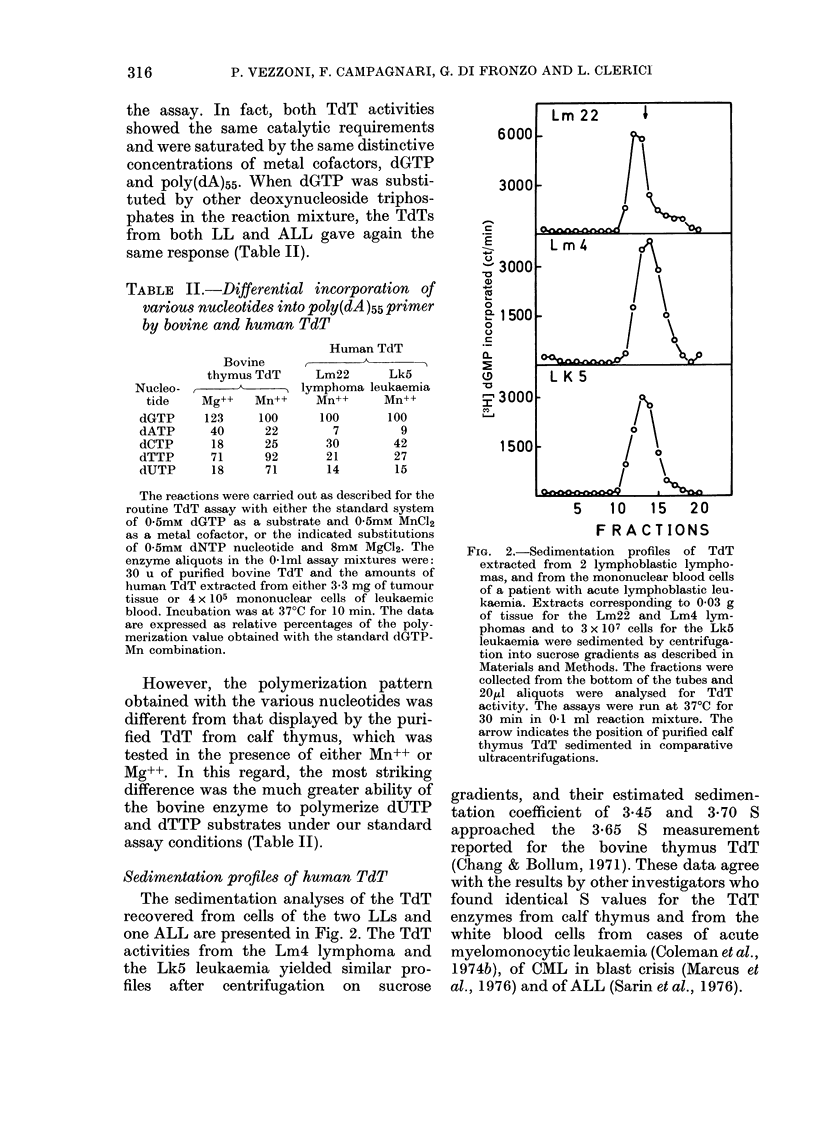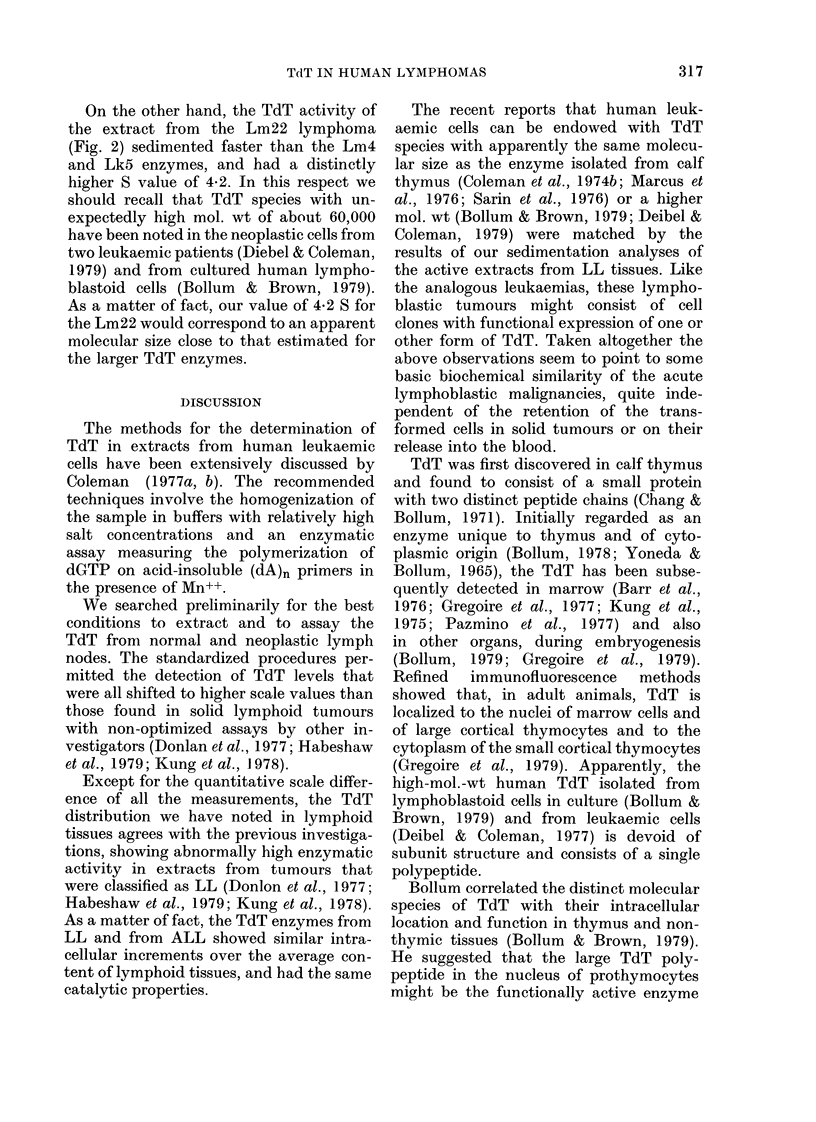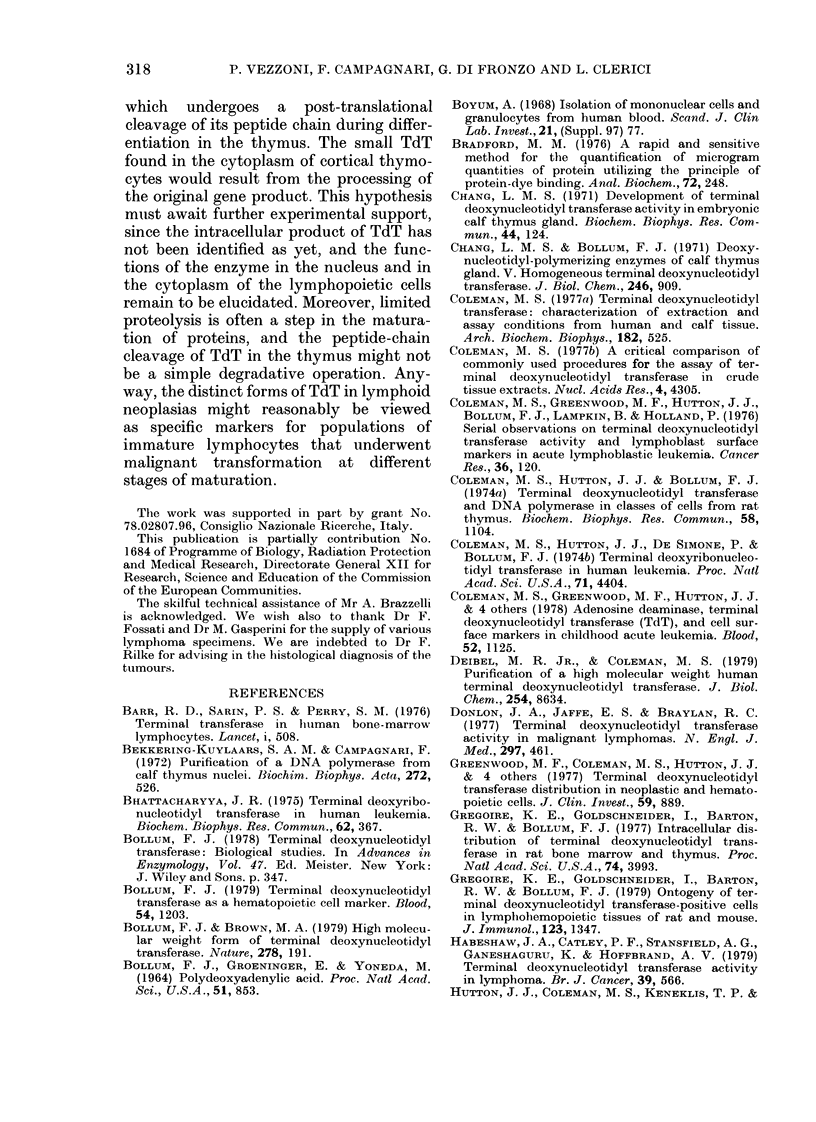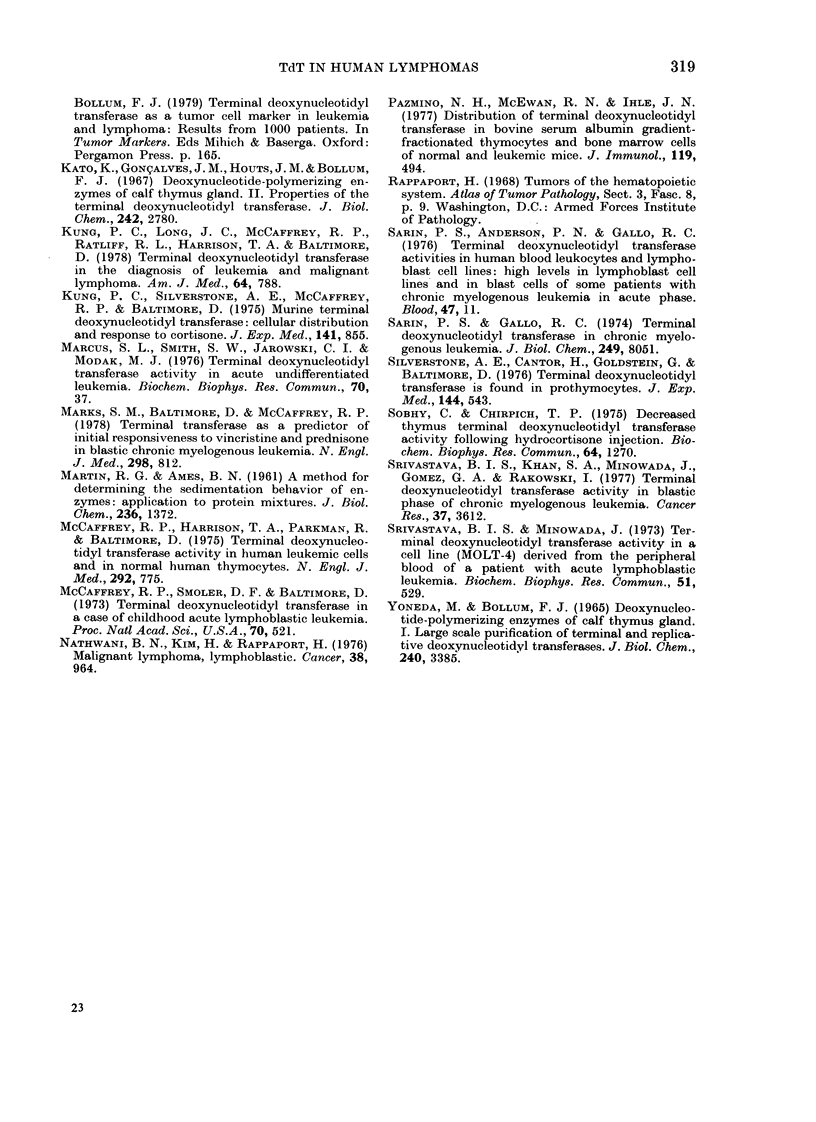# Terminal deoxynucleotidyl transferase in human lymphomas: possible existence of forms with high and low molecular weights.

**DOI:** 10.1038/bjc.1981.49

**Published:** 1981-03

**Authors:** P. Vezzoni, F. Campagnari, G. Di Fronzo, L. Clerici

## Abstract

Optimized methods for extraction and enzyme assay in crude tissue preparations were used to determine the amounts of terminal deoxnucleotidyl transferase (TdT) in malignant lymphomas. The TdT concentration was increased only in lymphoblastic lymphomas (LL) and was as high in these tumours as in the white blood cells from untreated patients with acute lymphoblastic leukaemia (ALL). The enzymes extracted from such lymphomas and from the leukaemic lymphoblasts had the same properties. Moreover, forms of TdT with low and high mol. wt were found in the LL tumours, similar to other reports of TdT-positive leukaemias. The overall study points at some basic biochemical identity of certain lymphoblastic malignancies, irrespective of whether the transformed cells are in solid tumours or are disseminated in the blood.


*Br. J. Cancer* (1981) **43**, 312

### TERMINAL DEOXYNUCLEOTIDYL TRANSFERASE IN HUMAN LYMPHOMAS: POSSIBLE EXISTENCE OF FORMS WITH HIGH AND LOW MOLECULAR WEIGHTS

P. VEZZONI†, F. CAMPAGNARI‡*, G. DI FRONZO† AND L. CLERICI‡

*From the †Laboratorio di Farmacocinetica Clinica, Istituto Nazionale per lo Studio e la Cura dei Tumori,* 20133 *Milano, and the ‡Laboratory of Biochemistry, Biology Group D.G. XII-Ispra, C.E.C., Joint Research Centre,* 21020 *Ispra (VA), Italy*

Received 22 September 1980 Accepted 26 November 1980

**Summary.—Optimized methods for extraction and enzyme assay in crude tissue preparations were used to determine the amounts of terminal deoxinucleotidyl transferase (TdT) in malignant lymphomas. The TdT concentration was increased only in lymphoblastic lymphomas (LL) and was as high in these tumours as in the white blood cells from untreated patients with acute lymphoblastic leukaemia (ALL). The enzymes extracted from such lymphomas and from the leukaemic lymphoblasts had the same properties. Moreover, forms of TdT with low and high mol. wt were found in the LL tumours, similar to other reports of TdT-positive leukaemias.**

**The overall study points at some basic biochemical identity of certain lymphoblastic malignancies, irrespective of whether the transformed cells are in solid tumours or are disseminated in the blood.**

THE ENZYME TERMINAL deoxynucleotidyl transferase (TdT) catalyzes the random condensation of deoxynucleotidyl residues on the 3′-OH termini of single-stranded DNA and of oligodeoxynucleotides (Bollum *et al.*, 1964; Chang & Bollum, 1971; Kato *et al.*, 1967; Yoneda & Bollum, 1965). TdT has a restricted distribution in nature, and is abundant only in the thymus of higher animals (Bollum, 1978), but it can be detected also in the marrow (Barr *et al.*, 1976; Gregoire *et al.*, 1977; Kung *et et.*, 1975; Pazmino *et al.*, 1977) and transiently in the spleen (Gregoire *et al.*, 1979). The enzyme is expressed in undifferentiated clones of migrating lymphoid cells that will finally evolve to T lymphocytes in the thymus (Bollum, 1979; Gregoire *et al.*, 1979; Silverstone *et al.*, 1976).

A general interest in TdT arose in 1973 after the reported presence of the enzyme in the neoplastic cells of acute lymphatic leukaemia (ALL) (McCaffrey *et al.*, 1973; Srivastava & Minowada, 1973). Subsequently, TdT was found in the white blood cells from individuals with chronic myelogenous leukaemia (CML) during the blastic phase (Bhattacharyya, 1975; Sarin & Gallo, 1974; Srivastava *et al.*, 1977) and in the blast cells from patients with acute undifferentiated leukaemia (AUL) (Kung *et al.*, 1978; Marcus *et al.*, 1976). The data gained to date (Coleman *et al.*, 1976, 1978; Greenwood *et al.*, 1977; Hutton *et al.*, 1979; Kung *et al.*, 1978; McCaffrey *et al.*, 1975; Sarin *et al.*, 1976) show that high levels of TdT are encountered in the immature cells from: most cases of ALL; 25–50% of CML in blast crisis; and a number of the so-called AUL. For these leukaemias, TdT is also a useful marker for the prediction of the response to therapy, since the cells with high TdT activity are often sensitive to cortisone (Kung *et al.*, 1975; Sobhy & Chirpich, 1975).

* To whom requests for reprints should be addressed.

Solid lymphoid tumours have not been as extensively investigated with regard to TdT as leukaemia. Apparently, only the rare lymphoblastic lymphomas (LL) have raised levels of TdT (Donlon *et al.*, 1977; Habeshaw *et al.*, 1979; Kung *et al.*, 1978).

This paper complements the existing reports on the distribution of TdT in different lymphomas. It confirms that the enzyme is functionally expressed in the LL tissue and it shows that the intracellular concentration of TdT in these tumours can be as high as in the blasts in peripheral blood from ALL patients. We have also found either small or large mol.-wt forms of TdT in the LL tumours, similar to the enzymes of low (Coleman *et al.*, 1974*b*; Marcus *et al.*, 1976; Sarin *et al.*, 1976) and high mol. wts (Deibel & Coleman, 1979) that had been described in TdT$^+$ leukaemias.

### MATERIALS AND METHODS

*Patients.*—Neoplastic lymphoid tissues from 31 males and 18 females of ages ranging from 5–77, as well as non-neoplastic lymph nodes from 8 patients, were analysed. The lymphoma samples were from lymph nodes in 47 cases and from mediastinal masses in 2. They were obtained directly from the operating room after surgery, immediately frozen and stored at −70°C until use. For the sake of comparison, white mononucleated cells of peripheral blood from 3 patients with TdT$^+$ leukaemias were also included in the study. Solid lymphoid tumours were classified histologically as proposed by Rappaport (1968) with the additional criteria introduced by Nathwani *et al.* (1976) for the definition of LL.

*Chemicals and reagents.*—Radioactive deoxynucleotides were purchased from Radiochemical Centre, Amersham. Unlabelled deoxynucleotides were from PL Biochemicals. Poly$(dA)_{55}$ was prepared enzymatically in our laboratory *via* the end addition reaction catalysed by purified bovine TdT in the presence of an oligo$(dA)_6$ initiator and dATP-Mg substrates (Kato *et al.*, 1967).

*Tissue and cell extracts.*—All procedures were carried out at 2°C. The frozen tissues were thawed, rinsed with saline, minced finely and suspended in 5 volumes of 0·25M potassium phosphate (pH 7·2). The mononucleated cells isolated from heparinized blood of leukaemia patients by centrifugation on Ficoll gradients (Boyum, 1968) were dispersed in the same phosphate buffer at a concentration of $2 \times 10^8$ cells/ml. The tissue and cell samples were disrupted in a Dounce homogenizer and the homogenates were spun at 100,000 *g* for 60 min. The supernatants obtained after centrifugation were considered as the crude extracts. Proteins were measured by the method of Bradford (1976), using human gamma-globulin as standard.

*Assays of TdT.*—The activity of human TdT was determined by measuring the induced polymerization of dGTP on an acid-insoluble $(dA)_n$ initiator as suggested by the group of Bollum for optimizing the enzyme assay in crude cell extracts (Chang, 1971; Chang & Bollum, 1971; Coleman *et al.*, 1974*a*, 1976). Care was taken to choose reaction conditions ensuring maximal rates of catalysis. The composition of the standard assay was guided by the kinetic data obtained experimentally with an active lymphoma extract, and is presented under Results. The method finally adopted uses $Mn^{++}$ instead of $Mg^{++}$ as metal cofactor and is similar to that proposed by Coleman (1977*a*, *b*) for studying human leukaemias. One unit of TdT is the amount polymerizing 1 nmol of nucleotide material in 1 h. TdT activity is expressed in terms of units/0·1 g of tissue, a mass estimated as equivalent to $10^8$ cells (Donlon *et al.*, 1977).

The TdT activity from calf thymus, purified according to the method of Yoneda & Bollum (1965), was assayed for comparison with the human TdT in our standard incubation mixture. Parallel assays were run also with 8mM $Mg^{++}$ as the divalent metal cofactor.

*Ultracentrifugation analysis on sucrose gradients.*—Sedimentation analysis of TdT activity in the tissue and cell extracts was carried out by centrifugation on sucrose gradients according to Martin & Ames (1961). Linear gradients from 5–20% sucrose in 50mM Tris–HCl (pH 8·0), 500mM NaCl, 1mM EDTA were prepared in the cellulose nitrate tubes of the SW 50·1 rotor of a Beckman L8-70 ultracentrifuge, and were overlaid with 0·2 ml aliquots of crude extracts. The gradients were spun for 18 h at 40,000 rev/min at 2°C. After centrifugation, serial fractions were collected from the bottom of the tubes and analysed for TdT activity. Bovine

FIG. 1.—Requirements for the assay of TdT in crude extracts from human lymphoma: saturation curves for primer, substrate and metal cofactors. The assay mixtures contained an active extract from the Lm4 lymphoma, the standard reactants and the indicated amounts of poly$(dA)_{55}$ primer, dGTP substrate and divalent metal cofactors. The data refer to initial polymerization rates expressed as deoxynucleotides incorporated into the acid-insoluble primer in 1 h at 37°C. The enzyme aliquots per ml of the final incubation mixtures corresponded to the TdT extracted from 0·03 g of tumour tissue.

serum albumin and chymotrypsinogen were used as markers for the determination of the sedimentation coefficient, S.

### RESULTS

### *Conditions of the assay*

The catalytic requirements of human TdT were preliminarily determined using an active extract from the lymphoblastic lymphoma Lm4 as a source of the enzyme. The relevant data are presented in Fig. 1.

The TdT activity was almost saturated by 7·5μM poly$(dA)_{55}$ as 3′-OH termini (panel a), and this was taken into account for defining 10μM poly$(dA)_{55}$ as the optimal concentration of initiator in the standard assay.

The rates of TdT reaction increased almost linearly on raising the concentration of dGTP substrate up to 0·2 mM, reached a maximum in the range of 0·2 to 1mM dGTP, and declined, because of inhibition, at higher levels (panel b).

The curves obtained for the saturation of TdT with $Mg^{++}$ and $Mn^{++}$ as metal cofactors are shown in panels c and d of Fig. 1, respectively. In contrast to the bovine TdT, the human enzyme clearly preferred $Mn^{++}$ to $Mg^{++}$ for active catalysis with a $(dA)_n$ initiator as first noted by Bhattacharyya (1975). In fact the Lm4 TdT became practically saturated at 0·2–1·0mM $Mn^{++}$, *i.e.* in a range one order of magnitude lower than the one needed by $Mg^{++}$ for optimal enzymatic activity. Moreover, the maximal reaction rates obtained in the presence of $Mn^{++}$ were almost 3-fold higher than those observed with saturating $Mg^{++}$ concentrations. This led us to choose 0·5mM $Mn^{++}$ as the metal cofactor in the standard assay.

The adopted mixture for the standard assay contained: 0·2M K-cacodylate (pH 7·0), 0·5mM $MnCl_2$, 0·5mM [$^3$H]dGTP ($10^4$ ct/min/nmol), 10μM poly$(dA)_{55}$ as 3′-OH and 20 μl of crude extract in a final volume of 100 μl. The reaction was run at 37°C for up to 1 h, the time kinetics always being linear for 15 min and for up to 60 min in many instances. Aliquots (20 μl) of the assay mixture were drawn at various intervals of time, spotted on discs of glass-fibre paper (Whatman GF/C) and processed for the determination of radioactivity in the acid-insoluble material, as previously described (Bekkering-Kuylaars & Campagnari, 1972).

### *Distribution of TdT activity in lymphomas*

Table I shows the various types of lymphoid tissues under study, and lists histological diagnosis, number of cases and the TdT concentrations. Most of the patients did not receive antiblastic therapy before the biopsy. The non-neoplastic lymph nodes taken as controls had a mean TdT content of 1·3 u/0·1 g of tissue, with values ranging from 0·5 to 2·7 times the mean.

The only group of lymphomas displaying high TdT activity was that of the

TABLE I.—*Distribution of TdT in lymphoid cells*

| | No. of cases | TdT/0·1 g of tissue or $10^8$ cells: Means or single values | Ranges |
|---|---|---|---|
| *Positive cells* | | | |
| Lymphoblastic lymphoma (LL) | | | |
| Untreated | 4 | 455 | 304–650 |
| Under drug treatment | 1 | 24 | — |
| Acute lymphoblastic leukemia (ALL) | 2 | 205; 225 | — |
| Chronic myelogenous leukemia, blast crisis (CML) | 1 | 1414 | — |
| *Negative cells* | | | |
| Hodgkin's disease | | | |
| Nodular sclerosis | 15 | 0·88 | 0·29–2·01 |
| Mixed cellularity | 2 | 0·75–1·02 | — |
| Lymphocytic predominance | 2 | 0·20; 3·60 | — |
| Non-Hodgkin's lymphoma | | | |
| Diffuse hystiocytic | 12 | 1·10 | 0·23–1·89 |
| Diffuse lymphocytic, well differentiated | 2 | 0·61; 0·65 | — |
| Diffuse lymphocytic, poorly differentiated | 3 | 1·12 | 0·85–1·33 |
| Diffuse mixed | 3 | 1·73 | 1·21–2·26 |
| Nodular lymphocytic, poorly differentiated | 3 | 0·94 | 0·53–1·20 |
| Burkitt's | 2 | 1·30; 2·40 | — |
| Non-neoplastic lymph nodes | 8 | 1·29 | 0·67–3·59 |

lymphoblastic type, LL. The neoplastic cells of the 4 untreated patients with this disease had concentrations of TdT that were about two orders of magnitude higher than the values found in non-neoplastic lymph nodes and in other lymphomas, and 1·5 to 3-fold larger than those in the leucocytes of the 2 ALL patients. The very large amounts of TdT found in the LL tumours at diagnosis are similar to those found by others with the same type of enzyme assay in mono-nucleated cells from blood and marrow of patients with either untreated TdT+ leukaemias (Coleman, 1977*a*; Coleman *et al.*, 1978) or leukaemic dissemination of LL (Hutton *et al.*, 1979).

A slightly raised level of TdT was found in the neoplastic tissue of an individual affected by LL and successfully treated with cortisone and cytostatic drugs. The TdT concentration in this tumour was far below the ones detected in the untreated LLs. Obviously, the expression of TdT in LL cells might depend upon the course of the disease in response to drug and hormone treatments. Within this context, the monitoring of the TdT concentration in LL is a useful clinical index for both the development of the tumour and its response to therapy as already noted by others in the leukaemias (Coleman *et al.*, 1976; Marks *et al.*, 1978).

The TdT activities measured in the solid lymphoid tissues were in general 10 times larger than those found by previous investigators (Donlon *et al.*, 1977; Habeshaw *et al.*, 1979; Kung *et al.*, 1978). This refers to the LL tumours and to the other lymphomas with apparently normal enzyme content, but it applies also to non-neoplastic lymph nodes. The discrepancy must be related to methodological differences in the determination of the TdT activity in crude tissue extracts, and it will be commented upon under Discussion.

*Comparative study of the TdT activities from lymphomas and leukaemias relative to purified bovine TdT*

The properties of the TdT extracted from LL tissue and from ALL cells were compared. The enzymes from the two sources yielded similar responses to the changes of the reactant concentrations in the assay. In fact, both TdT activities showed the same catalytic requirements and were saturated by the same distinctive concentrations of metal cofactors, dGTP and poly(dA)$_{55}$. When dGTP was substituted by other deoxynucleoside triphosphates in the reaction mixture, the TdTs from both LL and ALL gave again the same response (Table II).

TABLE II.—*Differential incorporation of various nucleotides into poly(dA)$_{55}$ primer by bovine and human TdT*

| Nucleotide | Bovine thymus TdT | | Human TdT: Lm22 lymphoma | Human TdT: Lk5 leukaemia |
|---|---|---|---|---|
| | $Mg^{++}$ | $Mn^{++}$ | $Mn^{++}$ | $Mn^{++}$ |
| dGTP | 123 | 100 | 100 | 100 |
| dATP | 40 | 22 | 7 | 9 |
| dCTP | 18 | 25 | 30 | 42 |
| dTTP | 71 | 92 | 21 | 27 |
| dUTP | 18 | 71 | 14 | 15 |

The reactions were carried out as described for the routine TdT assay with either the standard system of 0·5mM dGTP as a substrate and 0·5mM $MnCl_2$ as a metal cofactor, or the indicated substitutions of 0·5mM dNTP nucleotide and 8mM $MgCl_2$. The enzyme aliquots in the 0·1ml assay mixtures were: 30 u of purified bovine TdT and the amounts of human TdT extracted from either 3·3 mg of tumour tissue or $4 \times 10^5$ mononuclear cells of leukaemic blood. Incubation was at 37°C for 10 min. The data are expressed as relative percentages of the polymerization value obtained with the standard dGTP-Mn combination.

However, the polymerization pattern obtained with the various nucleotides was different from that displayed by the purified TdT from calf thymus, which was tested in the presence of either $Mn^{++}$ or $Mg^{++}$. In this regard, the most striking difference was the much greater ability of the bovine enzyme to polymerize dUTP and dTTP substrates under our standard assay conditions (Table II).

*Sedimentation profiles of human TdT*

The sedimentation analyses of the TdT recovered from cells of the two LLs and one ALL are presented in Fig. 2. The TdT activities from the Lm4 lymphoma and the Lk5 leukaemia yielded similar profiles after centrifugation on sucrose gradients, and their estimated sedimentation coefficient of 3·45 and 3·70 S approached the 3·65 S measurement reported for the bovine thymus TdT (Chang & Bollum, 1971). These data agree with the results by other investigators who found identical S values for the TdT enzymes from calf thymus and from the white blood cells from cases of acute myelomonocytic leukaemia (Coleman *et al.*, 1974*b*), of CML in blast crisis (Marcus *et al.*, 1976) and of ALL (Sarin *et al.*, 1976).

FIG. 2.—Sedimentation profiles of TdT extracted from 2 lymphoblastic lymphomas, and from the mononuclear blood cells of a patient with acute lymphoblastic leukaemia. Extracts corresponding to 0·03 g of tissue for the Lm22 and Lm4 lymphomas and to $3 \times 10^7$ cells for the Lk5 leukaemia were sedimented by centrifugation into sucrose gradients as described in Materials and Methods. The fractions were collected from the bottom of the tubes and 20μl aliquots were analysed for TdT activity. The assays were run at 37°C for 30 min in 0·1 ml reaction mixture. The arrow indicates the position of purified calf thymus TdT sedimented in comparative ultracentrifugations.

On the other hand, the TdT activity of the extract from the Lm22 lymphoma (Fig. 2) sedimented faster than the Lm4 and Lk5 enzymes, and had a distinctly higher S value of 4·2. In this respect we should recall that TdT species with unexpectedly high mol. wt of about 60,000 have been noted in the neoplastic cells from two leukaemic patients (Diebel & Coleman, 1979) and from cultured human lymphoblastoid cells (Bollum & Brown, 1979). As a matter of fact, our value of 4·2 S for the Lm22 would correspond to an apparent molecular size close to that estimated for the larger TdT enzymes.

### DISCUSSION

The methods for the determination of TdT in extracts from human leukaemic cells have been extensively discussed by Coleman (1977*a*, *b*). The recommended techniques involve the homogenization of the sample in buffers with relatively high salt concentrations and an enzymatic assay measuring the polymerization of dGTP on acid-insoluble $(dA)_n$ primers in the presence of $Mn^{++}$.

We searched preliminarily for the best conditions to extract and to assay the TdT from normal and neoplastic lymph nodes. The standardized procedures permitted the detection of TdT levels that were all shifted to higher scale values than those found in solid lymphoid tumours with non-optimized assays by other investigators (Donlan *et al.*, 1977; Habeshaw *et al.*, 1979; Kung *et al.*, 1978).

Except for the quantitative scale difference of all the measurements, the TdT distribution we have noted in lymphoid tissues agrees with the previous investigations, showing abnormally high enzymatic activity in extracts from tumours that were classified as LL (Donlon *et al.*, 1977; Habeshaw *et al.*, 1979; Kung *et al.*, 1978). As a matter of fact, the TdT enzymes from LL and from ALL showed similar intracellular increments over the average content of lymphoid tissues, and had the same catalytic properties.

The recent reports that human leukaemic cells can be endowed with TdT species with apparently the same molecular size as the enzyme isolated from calf thymus (Coleman *et al.*, 1974*b*; Marcus *et al.*, 1976; Sarin *et al.*, 1976) or a higher mol. wt (Bollum & Brown, 1979; Deibel & Coleman, 1979) were matched by the results of our sedimentation analyses of the active extracts from LL tissues. Like the analogous leukaemias, these lymphoblastic tumours might consist of cell clones with functional expression of one or other form of TdT. Taken altogether the above observations seem to point to some basic biochemical similarity of the acute lymphoblastic malignancies, quite independent of the retention of the transformed cells in solid tumours or on their release into the blood.

TdT was first discovered in calf thymus and found to consist of a small protein with two distinct peptide chains (Chang & Bollum, 1971). Initially regarded as an enzyme unique to thymus and of cytoplasmic origin (Bollum, 1978; Yoneda & Bollum, 1965), the TdT has been subsequently detected in marrow (Barr *et al.*, 1976; Gregoire *et al.*, 1977; Kung *et al.*, 1975; Pazmino *et al.*, 1977) and also in other organs, during embryogenesis (Bollum, 1979; Gregoire *et al.*, 1979). Refined immunofluorescence methods showed that, in adult animals, TdT is localized to the nuclei of marrow cells and of large cortical thymocytes and to the cytoplasm of the small cortical thymocytes (Gregoire *et al.*, 1979). Apparently, the high-mol.-wt human TdT isolated from lymphoblastoid cells in culture (Bollum & Brown, 1979) and from leukaemic cells (Deibel & Coleman, 1977) is devoid of subunit structure and consists of a single polypeptide.

Bollum correlated the distinct molecular species of TdT with their intracellular location and function in thymus and non-thymic tissues (Bollum & Brown, 1979). He suggested that the large TdT polypeptide in the nucleus of prothymocytes might be the functionally active enzyme which undergoes a post-translational cleavage of its peptide chain during differentiation in the thymus. The small TdT found in the cytoplasm of cortical thymocytes would result from the processing of the original gene product. This hypothesis must await further experimental support, since the intracellular product of TdT has not been identified as yet, and the functions of the enzyme in the nucleus and in the cytoplasm of the lymphopoietic cells remain to be elucidated. Moreover, limited proteolysis is often a step in the maturation of proteins, and the peptide-chain cleavage of TdT in the thymus might not be a simple degradative operation. Anyway, the distinct forms of TdT in lymphoid neoplasias might reasonably be viewed as specific markers for populations of immature lymphocytes that underwent malignant transformation at different stages of maturation.

The work was supported in part by grant No. 78.02807.96, Consiglio Nazionale Ricerche, Italy.

This publication is partially contribution No. 1684 of Programme of Biology, Radiation Protection and Medical Research, Directorate General XII for Research, Science and Education of the Commission of the European Communities.

The skilful technical assistance of Mr A. Brazzelli is acknowledged. We wish also to thank Dr F. Fossati and Dr M. Gasperini for the supply of various lymphoma specimens. We are indebted to Dr F. Rilke for advising in the histological diagnosis of the tumours.

### REFERENCES

Barr, R. D., Sarin, P. S. & Perry, S. M. (1976) Terminal transferase in human bone-marrow lymphocytes. *Lancet*, i, 508.

Bekkering-Kuylaars, S. A. M. & Campagnari, F. (1972) Purification of a DNA polymerase from calf thymus nuclei. *Biochim. Biophys. Acta*, **272**, 526.

Bhattacharyya, J. R. (1975) Terminal deoxyribonucleotidyl transferase in human leukemia. *Biochem. Biophys. Res. Commun.*, **62**, 367.

Bollum, F. J. (1978) Terminal deoxynucleotidyl transferase: Biological studies. In *Advances in Enzymology, Vol. 47*. Ed. Meister. New York: J. Wiley and Sons. p. 347.

Bollum, F. J. (1979) Terminal deoxynucleotidyl transferase as a hematopoietic cell marker. *Blood*, **54**, 1203.

Bollum, F. J. & Brown, M. A. (1979) High molecular weight form of terminal deoxynucleotidyl transferase. *Nature*, **278**, 191.

Bollum, F. J., Groeninger, E. & Yoneda, M. (1964) Polydeoxyadenylic acid. *Proc. Natl Acad. Sci., U.S.A.*, **51**, 853.

Boyum, A. (1968) Isolation of mononuclear cells and granulocytes from human blood. *Scand. J. Clin Lab. Invest.*, **21**, (Suppl. 97) 77.

Bradford, M. M. (1976) A rapid and sensitive method for the quantification of microgram quantities of protein utilizing the principle of protein-dye binding. *Anal. Biochem.*, **72**, 248.

Chang, L. M. S. (1971) Development of terminal deoxynucleotidyl transferase activity in embryonic calf thymus gland. *Biochem. Biophys. Res. Commun.*, **44**, 124.

Chang, L. M. S. & Bollum, F. J. (1971) Deoxynucleotidyl-polymerizing enzymes of calf thymus gland. V. Homogeneous terminal deoxynucleotidyl transferase. *J. Biol. Chem.*, **246**, 909.

Coleman, M. S. (1977*a*) Terminal deoxynucleotidyl transferase: characterization of extraction and assay conditions from human and calf tissue. *Arch. Biochem. Biophys.*, **182**, 525.

Coleman, M. S. (1977*b*) A critical comparison of commonly used procedures for the assay of terminal deoxynucleotidyl transferase in crude tissue extracts. *Nucl. Acids Res.*, **4**, 4305.

Coleman, M. S., Greenwood, M. F., Hutton, J. J., Bollum, F. J., Lampkin, B. & Holland, P. (1976) Serial observations on terminal deoxynucleotidyl transferase activity and lymphoblast surface markers in acute lymphoblastic leukemia. *Cancer Res.*, **36**, 120.

Coleman, M. S., Hutton, J. J. & Bollum, F. J. (1974*a*) Terminal deoxynucleotidyl transferase and DNA polymerase in classes of cells from rat thymus. *Biochem. Biophys. Res. Commun.*, **58**, 1104.

Coleman, M. S., Hutton, J. J., De Simone, P. & Bollum, F. J. (1974*b*) Terminal deoxyribonucleotidyl transferase in human leukemia. *Proc. Natl Acad. Sci. U.S.A.*, **71**, 4404.

Coleman, M. S., Greenwood, M. F., Hutton, J. J. & 4 others (1978) Adenosine deaminase, terminal deoxynucleotidyl transferase (TdT), and cell surface markers in childhood acute leukemia. *Blood*, **52**, 1125.

Deibel, M. R. Jr., & Coleman, M. S. (1979) Purification of a high molecular weight human terminal deoxynucleotidyl transferase. *J. Biol. Chem.*, **254**, 8634.

Donlon, J. A., Jaffe, E. S. & Braylan, R. C. (1977) Terminal deoxynucleotidyl transferase activity in malignant lymphomas. *N. Engl. J. Med.*, **297**, 461.

Greenwood, M. F., Coleman, M. S., Hutton, J. J. & 4 others (1977) Terminal deoxynucleotidyl transferase distribution in neoplastic and hematopoietic cells. *J. Clin. Invest.*, **59**, 889.

Gregoire, K. E., Goldschneider, I., Barton, R. W. & Bollum, F. J. (1977) Intracellular distribution of terminal deoxynucleotidyl transferase in rat bone marrow and thymus. *Proc. Natl Acad. Sci. U.S.A.*, **74**, 3993.

Gregoire, K. E., Goldschneider, I., Barton, R. W. & Bollum, F. J. (1979) Ontogeny of terminal deoxynucleotidyl transferase-positive cells in lymphohemopoietic tissues of rat and mouse. *J. Immunol.*, **123**, 1347.

Habeshaw, J. A., Catley, P. F., Stansfield, A. G., Ganeshaguru, K. & Hoffbrand, A. V. (1979) Terminal deoxynucleotidyl transferase activity in lymphoma. *Br. J. Cancer*, **39**, 566.

Hutton, J. J., Coleman, M. S., Keneklis, T. P. &

Bollum, F. J. (1979) Terminal deoxynucleotidyl transferase as a tumor cell marker in leukemia and lymphoma: Results from 1000 patients. In *Tumor Markers*. Eds Mihich & Baserga. Oxford: Pergamon Press. p. 165.

Kato, K., Gonçalves, J. M., Houts, J. M. & Bollum, F. J. (1967) Deoxynucleotide-polymerizing enzymes of calf thymus gland. II. Properties of the terminal deoxynucleotidyl transferase. *J. Biol. Chem.*, **242**, 2780.

Kung, P. C., Long, J. C., McCaffrey, R. P., Ratliff, R. L., Harrison, T. A. & Baltimore, D. (1978) Terminal deoxynucleotidyl transferase in the diagnosis of leukemia and malignant lymphoma. *Am. J. Med.*, **64**, 788.

Kung, P. C., Silverstone, A. E., McCaffrey, R. P. & Baltimore, D. (1975) Murine terminal deoxynucleotidyl transferase: cellular distribution and response to cortisone. *J. Exp. Med.*, **141**, 855.

Marcus, S. L., Smith, S. W., Jarowski, C. I. & Modak, M. J. (1976) Terminal deoxynucleotidyl transferase activity in acute undifferentiated leukemia. *Biochem. Biophys. Res. Commun.*, **70**, 37.

Marks, S. M., Baltimore, D. & McCaffrey, R. P. (1978) Terminal transferase as a predictor of initial responsiveness to vincristine and prednisone in blastic chronic myelogenous leukemia. *N. Engl. J. Med.*, **298**, 812.

Martin, R. G. & Ames, B. N. (1961) A method for determining the sedimentation behavior of enzymes: application to protein mixtures. *J. Biol. Chem.*, **236**, 1372.

McCaffrey, R. P., Harrison, T. A., Parkman, R. & Baltimore, D. (1975) Terminal deoxynucleotidyl transferase activity in human leukemic cells and in normal human thymocytes. *N. Engl. J. Med.*, **292**, 775.

McCaffrey, R. P., Smoler, D. F. & Baltimore, D. (1973) Terminal deoxynucleotidyl transferase in a case of childhood acute lymphoblastic leukemia. *Proc. Natl Acad. Sci., U.S.A.*, **70**, 521.

Nathwani, B. N., Kim, H. & Rappaport, H. (1976) Malignant lymphoma, lymphoblastic. *Cancer*, **38**, 964.

Pazmino, N. H., McEwan, R. N. & Ihle, J. N. (1977) Distribution of terminal deoxynucleotidyl transferase in bovine serum albumin gradient-fractionated thymocytes and bone marrow cells of normal and leukemic mice. *J. Immunol.*, **119**, 494.

Rappaport, H. (1968) Tumors of the hematopoietic system. *Atlas of Tumor Pathology*, Sect. 3, Fasc. 8, p. 9. Washington, D.C.: Armed Forces Institute of Pathology.

Sarin, P. S., Anderson, P. N. & Gallo, R. C. (1976) Terminal deoxynucleotidyl transferase activities in human blood leukocytes and lymphoblast cell lines: high levels in lymphoblast cell lines and in blast cells of some patients with chronic myelogenous leukemia in acute phase. *Blood*, **47**, 11.

Sarin, P. S. & Gallo, R. C. (1974) Terminal deoxynucleotidyl transferase in chronic myelogenous leukemia. *J. Biol. Chem.*, **249**, 8051.

Silverstone, A. E., Cantor, H., Goldstein, G. & Baltimore, D. (1976) Terminal deoxynucleotidyl transferase is found in prothymocytes. *J. Exp. Med.*, **144**, 543.

Sobhy, C. & Chirpich, T. P. (1975) Decreased thymus terminal deoxynucleotidyl transferase activity following hydrocortisone injection. *Biochem. Biophys. Res. Commun.*, **64**, 1270.

Srivastava, B. I. S., Khan, S. A., Minowada, J., Gomez, G. A. & Rakowski, I. (1977) Terminal deoxynucleotidyl transferase activity in blastic phase of chronic myelogenous leukemia. *Cancer Res.*, **37**, 3612.

Srivastava, B. I. S. & Minowada, J. (1973) Terminal deoxynucleotidyl transferase activity in a cell line (MOLT-4) derived from the peripheral blood of a patient with acute lymphoblastic leukemia. *Biochem. Biophys. Res. Commun.*, **51**, 529.

Yoneda, M. & Bollum, F. J. (1965) Deoxynucleotide-polymerizing enzymes of calf thymus gland. I. Large scale purification of terminal and replicative deoxynucleotidyl transferases. *J. Biol. Chem.*, **240**, 3385.